# Laminin-511 and -521-based matrices for efficient *ex vivo*-expansion of human limbal epithelial progenitor cells

**DOI:** 10.1038/s41598-017-04916-x

**Published:** 2017-07-11

**Authors:** Naresh Polisetti, Lydia Sorokin, Naoki Okumura, Noriko Koizumi, Shigeru Kinoshita, Friedrich E. Kruse, Ursula Schlötzer-Schrehardt

**Affiliations:** 1 Department of Ophthalmology, Universitätsklinikum Erlangen, Friedrich-Alexander-Universität Erlangen-Nürnberg, Erlangen, Germany; 2 Institute of Physiological Chemistry and Pathobiochemistry and Cells-in-Motion Cluster of Excellence, University of Muenster, Muenster, Germany; 3 Department of Biomedical Engineering, Faculty of Life and Medical Sciences, Doshisha University, Kyotanabe, Japan; 4 Department of Frontier Medical Science and Technology for Ophthalmology, Kyoto Prefectural University of Medicine, Kyoto, Japan

## Abstract

Optimization of culture conditions for human limbal epithelial stem/progenitor cells (LEPC) that incorporate the *in vivo* cell-matrix interactions are essential to enhance LEPC *ex vivo*-expansion and transplantation efficiency. Here, we investigate the efficacy of laminin (LN) isoforms preferentially expressed in the limbal niche as culture matrices for epithelial tissue engineering. Analyses of expression patterns of LN chains in the human limbal niche provided evidence for enrichment of LN-α2, -α3, -α5, -β1, -β2, -β3, -γ1, -γ2 and -γ3 chains in the limbal basement membrane, with LN-α5 representing a signature component specifically produced by epithelial progenitor cells. Recombinant human LN-521 and LN-511 significantly enhanced *in vitro* LEPC adhesion, migration and proliferation compared to other isoforms, and maintained phenotype stability. The bioactive LN-511-E8 fragment carrying only C-terminal domains showed similar efficacy as full-length LN-511. Functional blocking of α3β1 and α6β1 integrins suppressed adhesion of LEPC to LN-511/521-coated surfaces. Cultivation of LEPC on fibrin-based hydrogels incorporating LN-511-E8 resulted in firm integrin-mediated adhesion to the scaffold and well-stratified epithelial constructs, with maintenance of a progenitor cell phenotype in their (supra)basal layers. Thus, the incorporation of chemically defined LN-511-E8 into biosynthetic scaffolds represents a promising approach for xeno-free corneal epithelial tissue engineering for ocular surface reconstruction.

## Introduction

Limbal epithelial stem/progenitor cells (LEPC) are located in the basal layer of the epithelium at the corneoscleral limbus and are responsible for homeostasis of the corneal epithelium, which is an important prerequisite for corneal transparency and visual function^1, 2^. Any damage or injury to this stem/progenitor cell reservoir and/or destruction of its niche microenvironment can lead to corneal neovascularization, chronic inflammation, and stromal scarring associated with corneal opacity and loss of vision^3–5^. Transplantation of *ex vivo-* expanded LEPC on amniotic membrane or fibrin gels is an established therapeutic strategy to regenerate the damaged corneal surface in patients with limbal stem cell deficiency (LSCD)^6–9^. Since its introduction in 1997^10^, cultured limbal epithelial transplantation (CLET) has been applied in various clinical centres with follow-up periods of more than 15 years^8, 11–14^. Despite many variables between studies (regarding inclusion/exclusion criteria, culture methods, transplantation techniques, and clinical outcome measures), long-term engraftment of autologous cultivated limbal epithelial cells has been shown to be good and the overall success rates of autologous CLET for unilateral LSCD with a follow-up period of at least 24 months were reported to amount to 72–76%^8, 15, 16^. In spite of these good clinical outcomes confirming CLET as an adequate therapy to successfully reconstruct the corneal surface in the majority of patients, long-term corneal regeneration in other series often proved less satisfactory due to recurrent mild neovascularisation of the corneal surface in many patients^17^. These complications might be caused by low quality of the graft or inadequate properties of transplanted progenitor cells^8, 11, 18^. A major hurdle in *in vitro* culturing of LEPC is that they readily differentiate, hampering their use for therapeutic applications^19, 20^. These limitations underscore the need for developing novel standardized LEPC culture techniques that ensure preservation of the stem/progenitor cell phenotype and function during cultivation and after transplantation.

*In vivo*, LEPC reside in a highly specialized and complex microenvironment that is known as the limbal niche, where stem cell quiescence, proliferation and differentiation are maintained in balance^21^. Besides several types of supporting niche cells that include melanocytes and mesenchymal stromal cells, the limbal niche comprises a specific extracellular matrix (ECM) composition^22^. Indeed, the ECM has been recognized as a crucial part of many stem cell niches mediating biophysical, mechanical and biochemical signals that regulate stem cell survival, proliferation and differentiation^23–26^. Laminins (LNs) are the best-described ECM constituents present in basement membranes (BM) of adult stem cell niches, where they influence cell behaviors, such as cell adhesion, differentiation, and phenotype stability. To date, five alpha (α1-5), four beta (β1-4), and three gamma (γ1-3) chains have been identified that can combine to form up to 16 heterotrimeric isoforms in mammalian tissues, showing tissue-specific distribution patterns^27–30^. We have previously shown that LN chains α1, α2, α5, β1, β2, γ1 and γ3 are preferentially localized to the BM of the limbal epithelium compared to that of the corneal epithelium, where LN chains α3, β3 and γ2 predominate^31^. Our work has further suggested that LEPC are anchored to LN-α2 and -α5 in their niche by expression of LN receptors α3β1 and α6β4 integrins^32^. The heterogeneity of LN isoform expression in BMs of ocular surface epithelia, which has been also reported by other groups^33–35^, suggests a functional role for LN-α2 and -α5 containing isoforms in the limbal stem cell niche.

Stem cell-based tissue engineering aims to mimic the native stem cell niche and to present the appropriate microenvironmental cues, including ECM components, in a controlled and reproducible fashion in order to maintain stem cell function within the graft^36, 37^. We have previously reported that the LN-332 isoform can serve as a suitable substrate to induce transdifferentiation of hair follicle stem cells into corneal epithelial-like cells in the presence of limbal fibroblast conditioned medium^38^. It has also been suggested that proteolytic fragments of LN-332 γ2 chain potentiate the outgrowth of limbal epithelial cells on intact amniotic membrane^39^, and that vitronectin adsorbed to therapeutic contact lenses support LEPC expansion^40^. Otherwise, little attention has been paid to the role of the ECM in LEPC *ex vivo*-expansion, and the effect of limbus-specific LNs on LEPC phenotype and function *in vitro* has not been investigated. We hypothesize that the LN isoforms that are specifically expressed in the limbal stem cell niche may be used as exogenous cues to promote *ex vivo*-expansion and maintenance of LEPC. In this study, we systematically analyze expression patterns of LN chains in LEPC clusters isolated by laser capture microdissection (LCM), in tissue sections and in cultured LEPC by quantitative RT-PCR (qPCR) and confocal microscopy, and test the effects of niche-specific recombinant LN isoforms on LEPC adhesion, migration, proliferation and differentiation *in vitro*. We also examine the efficacy of a recombinant human LN-511-E8 fragment^41, 42^ for epithelial tissue engineering using fibrin-based hydrogels as carrier. Our findings demonstrate that LN-α5 constitutes a signature BM component of the limbal niche and that LN-511 or -521 promote *ex vivo*-expansion and maintenance of LEPC. The C-terminal fragment of LN-511 (LN-511-E8 fragment) shows comparable efficacy as full-length LN-511 and supports the formation of well stratified epithelial constructs with maintenance of a progenitor cell phenotype. Thus, LN-511-E8 represents a chemically defined, xeno-free substrate for improved corneal epithelial tissue engineering and future clinical application.

## Results

### Expression of laminin chains in the limbal stem cell niche *in situ*

**Figure 1:**
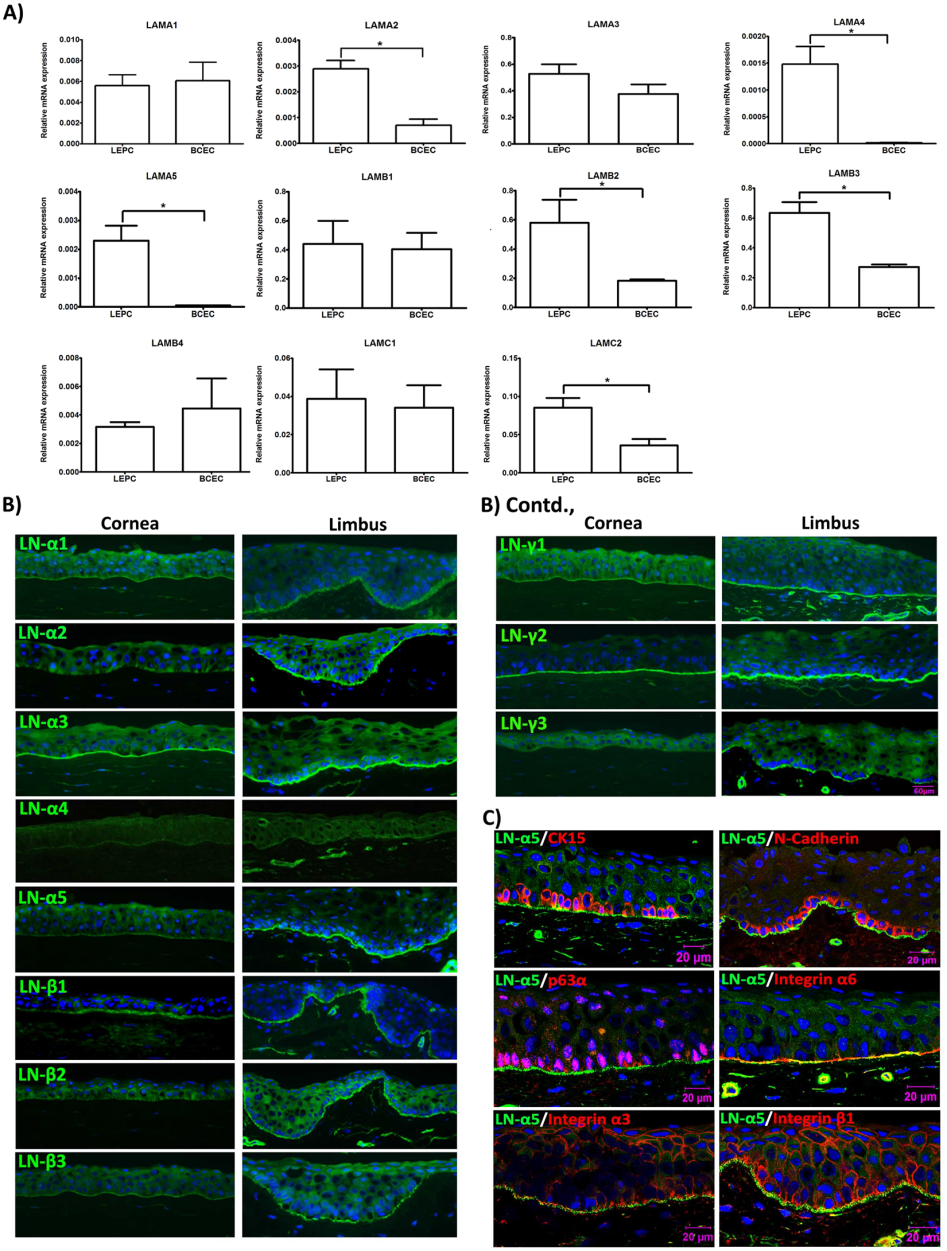
Expression of laminin chains in the limbal stem cell niche *in situ*. (**A**) Quantitative real-time polymerase chain reaction (qRT-PCR) primer assays showing higher expression levels of laminin α2 (LAMA2), α4 (LAMA4), α5 (LAMA5), β2 (LAMB2), β3 (LAMB3), and γ2 (LAMC2) in microdissected limbal epithelial stem/progenitor cell (LEPC) clusters compared with basal corneal epithelial cell (BCEC) populations; laminin α1 (LAMA1), α3 (LAMA3), β1 (LAMB1), β4 (LAMB4), γ1 (LAMC1) showed no differential expression patterns. Data are expressed as means (2^−ΔCT^ × 1,000) ± SEM (*n* = 5); *p < 0.05; Mann-Whitney *U* test. (**B**) Immunofluorescence analyses of corneoscleral tissue sections showing differential staining patterns of laminin α2, α5, β2, β3, γ2, and γ3, but similar staining patterns of laminin α1, α3, β1, and γ1 in the basement membranes of corneal and limbal epithelia; laminin α4 was largely negative in epithelial basement membranes. Nuclei are counterstained with DAPI (blue); scale bar = 60 µm. (**C**) Immunofluorescence double labeling of laminin (LN) α5 (green) and cytokeration (CK)15, N-Cadherin, p63α, integrin α6, integrin α3, and integrin β1 (red); nuclear counterstaining with DAPI (blue); scale bar = 20 µm.

Expression patterns of different LN chains were analyzed in LCM-dissected LEPC clusters, containing both epithelial stem/progenitor cells and associated niche cells^32^, and basal corneal epithelial cell (BCEC) populations by qPCR (n = 5). Quality control of amplified RNA and purity of dissected cell populations were as described previously^32^. All LN chains were expressed in both LEPC and BCEC populations except LN-γ3 (*LAMC3*). Expression levels of LN-α2 (*LAMA2*: 3.1 ± 0.6-fold; p = 0.02), LN-α4 (*LAMA4*: 98.3 ± 50.0-fold; p = 0.02), LN-α5 (*LAMA5:* 7.2 ± 4.6-fold; p = 0.01), LN-β2 (*LAMB2*: 3.0 ± 0.4-fold; p = 0.04), LN-β3 (*LAMB3*: 3.0 ± 0.8-fold; p = 0.02) and LN-γ2 (*LAMC2*: 3.0 ± 0.8-fold; p = 0.04) were significantly higher in LEPC compared with BCEC, whereas no differential expression patterns were observed for LN-α1 (*LAMA1*), -α3 (*LAMA3*), -β1 (*LAMB1*), -β4 (*LAMB4*), and -γ1 (*LAMC1*) (Fig. 1A). Of note, corneal epithelial expression levels of LN-α4 and –α5 were hardly above the detection threshold.

By immunohistochemistry using chain-specific antibodies (Table 1; n = 10), the LN-α2, -α3, -α5, -ß1, -ß2, -ß3, -γ1, and -γ2 chains were shown to be strongly expressed in the limbal BM, whereas LN-α1 and -γ3 chains were only weakly expressed (Fig. 1B). The LN-α4 chain was not detected in epithelial but only in vascular basement membranes (Fig. 1B). Antibodies against LN-ß4 were not available. The most pronounced *differential* expression patterns between limbal and corneal regions were observed for LN-α2, -α5, -ß2, and -γ3 chains, which could be hardly detected in the corneal BM (Fig. 1B).

**Table 1:** List of antibodies used.

Antibody (clone), Host species	Antibody concentration	Application	Antibody source [Ref.]
CD31 (WM59), mouse	15 µg/ml	Flow Cytometry	BD
CD34 (563), mouse	50 µg/ml	Flow Cytometry	BD
CD44 (G44-26), mouse	25 µg/ml	Flow Cytometry	BD
CD45 (HI30), mouse	50 µg/ml	Flow Cytometry	BD
CD73 (AD2), mouse	50 µg/ml	Flow Cytometry	BD
CD90 (5E10), mouse	100 µg/ml	Flow Cytometry	BD
CD105 (266), mouse	50 µg/ml	Flow Cytometry	BD
Mouse IgG1k	100 µg/ml	Flow Cytometry	BD
CD49c (Integrin α3) APC (P1B5), mouse	1.25 µg/ml	Flow Cytometry	eBioscience
CD49f (Integrin α6) APC (GoH3), rat	1.25 µg/ml	Flow Cytometry	eBioscience
CD29 (Integrin β1) FITC (TS2/16), mouse	2.5 µg/ml	Flow Cytometry	eBioscience
CD104 (Integrin β4) eFluor 660 (439-9B), rat	10 µg/ml	Flow Cytometry	eBioscience
IgG1 isotype FITC (IS5-21F5), mouse	2.5 µg/ml	Flow Cytometry	Miltenyi Biotec
IgG1 isotype APC (IS5-21F5), mouse	1.25–2.5 µg/ml	Flow Cytometry	Miltenyi Biotec
IgG2b isotype APC (141945), rat	1.25–10 µg/ml	Flow Cytometry	R&D Systems
Integrin α3 (ASC-1), mouse	20 µg/ml	Blocking	Millipore
Integrin α6 (NKI-GoH3), rat	20 µg/ml	Blocking	Millipore
Integrin β1 (P5D2), mouse	2.5 µg/ml	Blocking	R&D Systems
Cytokeratin 3 (AE5), mouse	1:100	Immunohistochemistry	Chemicon/Millipore
Cytokeratin 15 (LHK15), mouse	1:500	Immunohistochemistry	Abcam
Integrin α3 (P1B5), mouse	1:200	Immunohistochemistry	Dako
Integrin α6 (GoH3), rat	1:100	Immunohistochemistry	Chemicon/Millipore
Integrin β1 (HB1.1), mouse	1:500	Immunohistochemistry	Chemicon/Millipore
Ki-67 (SP6), rabbit	1:1000	Immunohistochemistry	Abcam
Laminin α1 (317), rabbit	1:500	Immunohistochemistry	L. Sorokin^63^
Laminin α2 (401), rabbit	1:500	Immunohistochemistry	L. Sorokin^63^
Laminin α3, rabbit	1:6000	Immunohistochemistry	R. Timpl/T. Sasaki
Laminin α4 (377), rabbit	1:2000	Immunohistochemistry	L. Sorokin^63^
Laminin α5 (405), rabbit	1:4000	Immunohistochemistry	L. Sorokin^63^
Laminin β1 (IIID9), mouse	undiluted	Immunohistochemistry	L. Sorokin^63^
Laminin β2 (409), rabbit	1:200	Immunohistochemistry	L. Sorokin^63^
Laminin β3, rabbit	1:6000	Immunohistochemistry	R. Timpl/T. Sasaki
Laminin γ1 (3E10), rat	undiluted	Immunohistochemistry	L. Sorokin^63^
Laminin γ2 (LE4-6), rabbit	1:4000	Immunohistochemistry	R. Timpl/T. Sasaki
Laminin γ3, rabbit	1:2000	Immunohistochemistry	R. Timpl/T. Sasaki
N-Cadherin (6G11), mouse	1:25	Immunohistochemistry	Dako
p63α, rabbit	1:100	Immunohistochemistry	Cell Signaling

Concurrent with the qPCR data, the immunohistochemical findings suggest that the human limbal niche is specifically enriched for LN-α2 and -α5 chains.These were found to co-localize with the progenitor cell markers cytokeratin (CK)15, N-cadherin, and p63α as well as with integrins α3, α6 and ß1 expressed by LEPC clusters in the basal epithelium at the limbus (data for LN-α5 is shown in Fig. 1C).

### Expression of laminin chains in limbal stem/progenitor and associated niche cells *in vitro*

**Figure 2:**
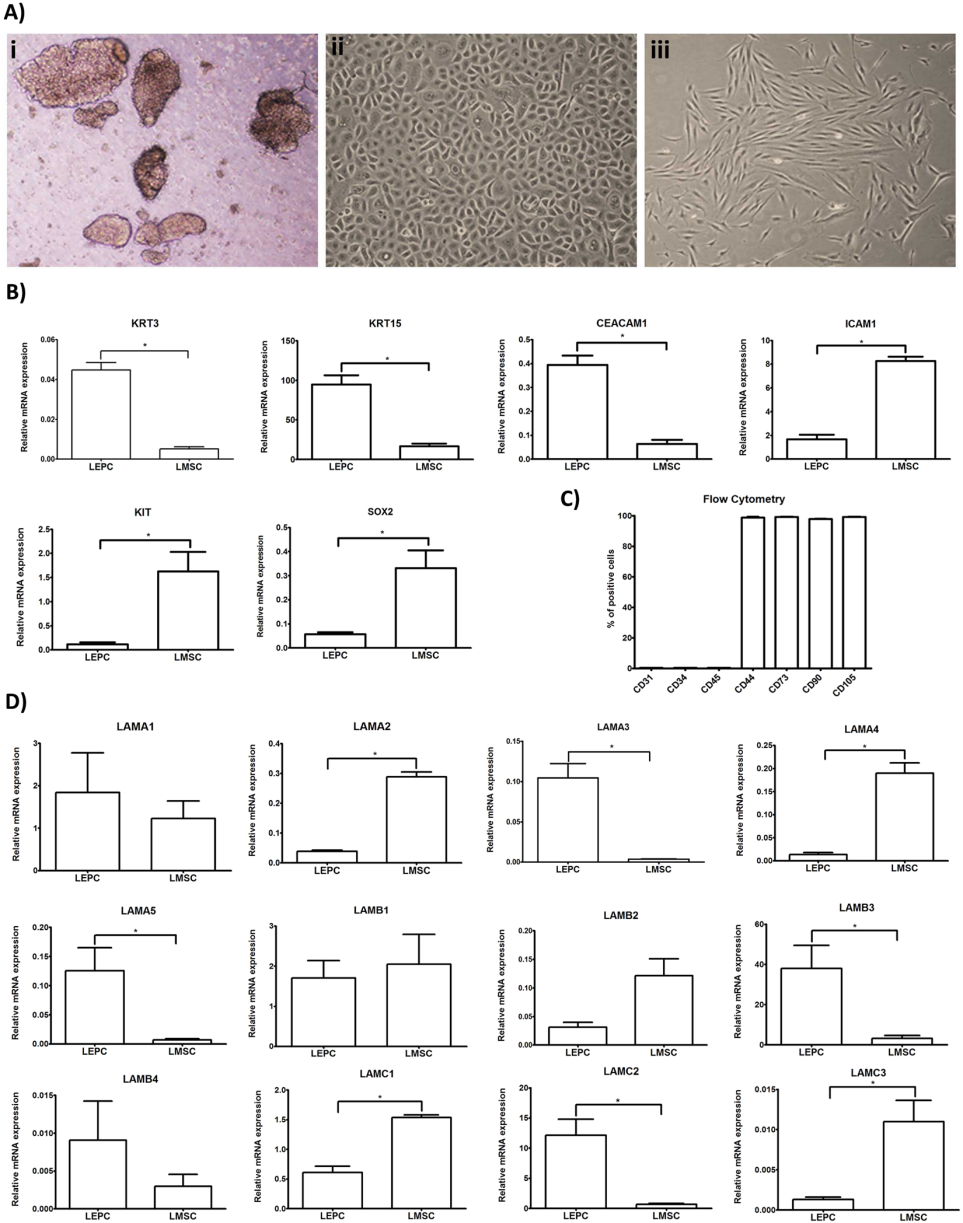
Expression of laminin chains in limbal stem/progenitor and associated niche cells *in vitro*. (**A**) Phase contrast images of (i) limbal epithelial cell clusters after collagenase digestion, (ii) cultured limbal epithelial progenitor cells, and (iii) associated mesenchymal stromal cells. (**B**) Quantitative real-time polymerase chain reaction (qRT-PCR) primer assays confirming differential expression of established epithelial *(KRT3*, *KRT15*, *CEACAM1*) and mesenchymal *(ICAM1*, *KIT*, *SOX2)* markers in cultured limbal epithelial progenitor cells (LEPC) compared with cultured limbal mesenchymal stromal cells (LMSC). Data are expressed as means (2^−ΔCT^ × 1,000) ± SEM (*n* = 5). (**C**) Flow cytometry analyses of cultured LMSC showing positive expression of CD44, CD73, CD90, and CD105, but negative expression of CD31, CD34, and CD45. Percentages (%) of positive cells are expressed as means ± SEM (*n* = 3). (**D**) qRT-PCR primer assays showing higher expression levels of laminin α3 (LAMA3), α5 (LAMA5), β3 (LAMB3), γ2 (LAMC2) in cultured LEPC, and higher expression levels of laminin α2 (LAMA2), α4 (LAMA4), β2 (LAMB2), γ1 (LAMC1), γ3 (LAMC3) in cultured LMSC; laminin α1 (LAMA1), β1 (LAMB1), and β4 (LAMB4) showed no differential expression patterns. Data are expressed as means (2^−ΔCT^ × 1,000) ± SEM (*n* = 5); *p < 0.05; Mann-Whitney *U* test. (Abbreviations: KRT, Keratin; CEACAM1, carcinoembryonic antigen-related cell adhesion molecule 1; ICAM1, intercellular cell adhesion molecule 1; Sox2, sex determining region Y-box 2; CD, cluster of differentiation).

To determine the relative contribution of LEPC and their associated stromal niche cells to expression of LN chains in the limbal niche, we established cultures of both LEPC and limbal mesenchymal stromal cells (LMSC) from collagenase-digested LEPC clusters (n = 5) as described previously (Fig. 2A)^32^. To verify the purity of cell populations, we analyzed the expression profiles of established corneal epithelial (progenitor) markers, such as cytokeratin 3 (KRT3) and 15 (KRT15) and carcinoembryonic antigen-related cell adhesion molecule 1 (CEACAM1), as well as mesenchymal (stem cell) markers, such as intercellular cell adhesion molecule 1 (ICAM1), sex determining region Y –box 2 (SOX2), and stem cell factor receptor (KIT) by qPCR primer assays (n = 5). As expected, LEPC populations showed significantly higher expression levels of KRT3 (10.3 ± 6.3-fold), KRT15 (5.6 ± 2.1-fold) and CEACAM (11.6 ± 4.5-fold), while LMSC populations showed higher expression levels of ICAM1 (5.4 ± 2.9-fold), SOX2 (5.0 ± 2.1-fold) and KIT (19.6 ± 10.2-fold) (p = 0.02) (Fig. 2B). LMSC were further characterized by flow cytometry (n = 3) with more than 90% of the cells expressing the mesenchymal stem cell markers CD73, CD90, CD105, and CD44 (Fig. 2C).

Differential gene expression analyses (n = 5) showed that LN-α3 (27.1 ± 17.3-fold; p = 0.02), -α5 (15.6 ± 10.4-fold; p = 0.02), -β3 (32.1 ± 27.3-fold; p = 0.02), and -γ2 (23.4 ± 11.9-fold; p = 0.03) chains were predominantly expressed in the LEPC population compared to LMSC, whereas LN-α2 (5.2 ± 2.6-fold; p = 0.02), -α4 (30.9 ± 21.5-fold; p = 0.02), -β2 (4.4 ± 0.8-fold; n.s.), -γ1 (2.7 ± 0.6-fold; p = 0.02), and -γ3 (8.4 ± 1.5-fold; p = 0.02) chains were predominantly expressed in LMSC compared to LEPC (Fig. 2D).

Together, these expression data obtained *in vivo* and *in vitro* suggest that LN-α5 constitutes a signature BM component of the limbal niche, which is endogenously produced by LEPC and becomes strongly enriched in the BM of the limbal niche.

### Effect of laminin isoforms on LEPC adhesion, migration, proliferation and differentiation

The cell-binding activities of LN isoforms are largely determined by α chains^27, 43^. As the availability of purified LN isoforms is limited, we performed *in vitro* tests of LEPC function using the two main LN-α5 chain containing isoforms, i.e. recombinant LN-511 and -521, as substrates, compared to isoforms containing LN-α1, -α2, -α3 or -α4 chains, i.e. recombinant LN-111, -211, -332, -411, and -421. Recombinant LN-511-E8 fragment which carries only C-terminal domains, where the main β1-integrin binding sites occur^41^, was tested in addition.

**Figure 3:**
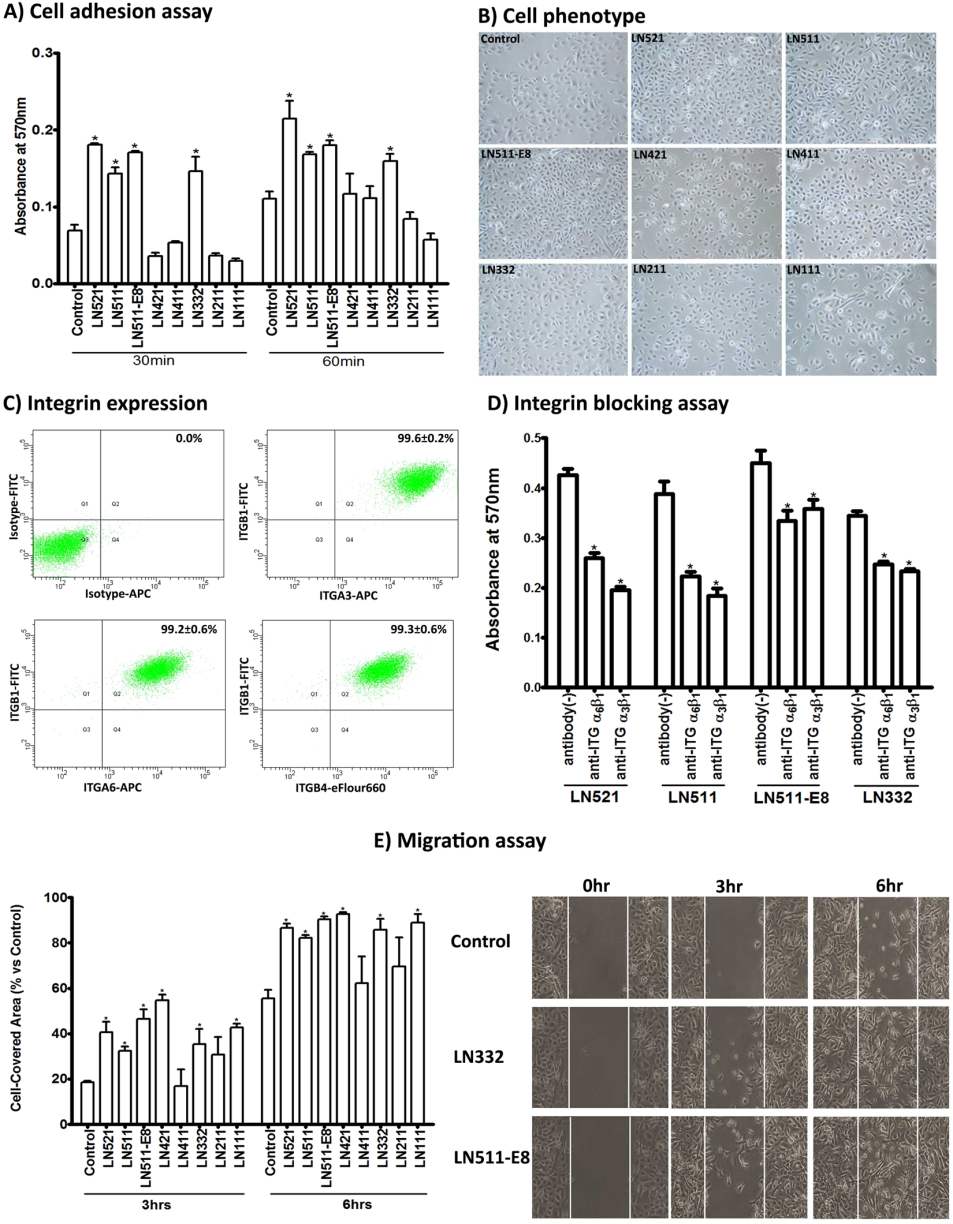
Effect of laminin isoforms on limbal progenitor cell adhesion and migration. (**A**) The effect of laminin (LN) isoforms on cell adhesion was tested by seeding limbal epithelial progenitor cells (LEPC) at a density of 50,000 cells/cm^2^ and spectrophotometric measurement of adherent cells 30 and 60 minutes after seeding. Data are expressed as means ± SEM (n = 4). (**B**) Phase contrast images of LEPC cultured on LN isoforms; magnification ×100. (**C**) Flow cytometry analyses of cultured LEPC showing expression of integrin α3 (ITGA3), integrin α6 (ITGA6), integrin β1 (ITGB1), and integrin β4 (ITGB4) on their surface. Percentages (%) of positive cells are expressed as means ± SEM percentage (%) (n = 3). (**D**) Functional blocking of integrin-mediated LEPC adhesion to LN-521, -511, -511-E8 and -332 was tested using neutralizing antibodies against integrin α3β1 and α6β1 60 minutes seeding. Data are expressed as means ± SEM (n = 4). (**E**) The effect of LN isoforms on LEPC migration was analyzed in two well-culture inserts with a defined cell-free gap and measurement of gap closure 3 and 6 hours after removal of the culture inserts. Data are expressed as means ± SEM (n = 3); *p < 0.05; Mann-Whitney *U* test.

The effect of the different LNs on cell adhesion was evaluated by determining the number of adherent LEPC on LN-coated (1.0 µg/cm^2^) culture wells at 30 and 60 min after seeding compared to uncoated tissue culture plates (n = 4). Coating with LN-521, LN-511, LN-511-E8 and LN-332 increased cell adhesion significantly over the uncoated control both after 30 and 60 min of incubation (Fig. 3A). Differences were significant (p = 0.02) for LN-521 (2.6-fold at 30 min and 1.9-fold at 60 min), LN-511 (2.0- and 1.5-fold), LN-511-E8 (2.5- and 1.6-fold) and LN-332 (2.1- and 1.4-fold). LN-421, LN-411, LN-221, and LN-111 did not support adhesion better than uncoated tissue culture plastic. Phase contrast microscopy showed a dense monolayer of epithelial cells on LN-521, -511, and 511-E8, but rather sparser layers on the other LN isoforms 24 hours after seeding (Fig. 3B).

LN-511/521 has been reported to bind to integrin α3β1 and α6β1, while LN-332 binds to integrin α6β4 on the cell membrane^44^. Because integrins α3β1and α6β4 have been previously reported to mediate anchorage of LEPC to their niche^32^, we analyzed the expression of these integrin subunits on the cell surface of cultured LEPC (n = 3) and evaluated the effect of integrin blocking on LEPC adhesion to LN-521, -511, -511-E8 and -332 (n = 4). As demonstrated by flow cytometry, almost 100% of LEPC expressed α3, α6, β1 and β4 integrins on their surface (Fig. 3C) and both neutralizing antibodies against α3β1 and α6β1 integrins significantly reduced adhesion of LEPC to LN-521 (α6β1: 39.1% (−1.6-fold); α3β1: 54.0% (−2.2-fold)), LN-511 (α6β1: 42.6% (−1.7-fold); α3β1: 52.5% (−2.1-fold)), LN-511-E8 (α6β1: 25.7% (−1.3-fold; α3β1: 20.4% (−1.2-fold)), and LN-332 (α6β1: 28.2% (−1.4-fold); α3β1: 32.2%(−1.5-fold)) as early as 1 hour after seeding (p = 0.02) (Fig. 3D). These findings indicate that LN-α5 and –α3 mediate LEPC adhesion through integrins α3β1 and α6β1 integrin binding.

To evaluate the effect of LN isoforms on LEPC migration, cells were plated on the different LN isoforms and gap closure following removal of a culture insert was analyzed 3 and 6 hours afterwards (n = 3). All LN isoforms, except LN-211 and LN-411, induced a significant increase in cell migration compared to uncoated controls at both time points (LN-521: 2.1- and 1.5-fold; LN-511: 1.7- and 1.4-fold; LN-511-E8: 2.5- and 1.6-fold; LN-421: 2.9- and 1.6-fold; LN-332: 1.8- and 1.4-fold; LN-111: 2.3- and 1.5-fold) (p = 0.02) (Fig. 3E).

**Figure 4:**
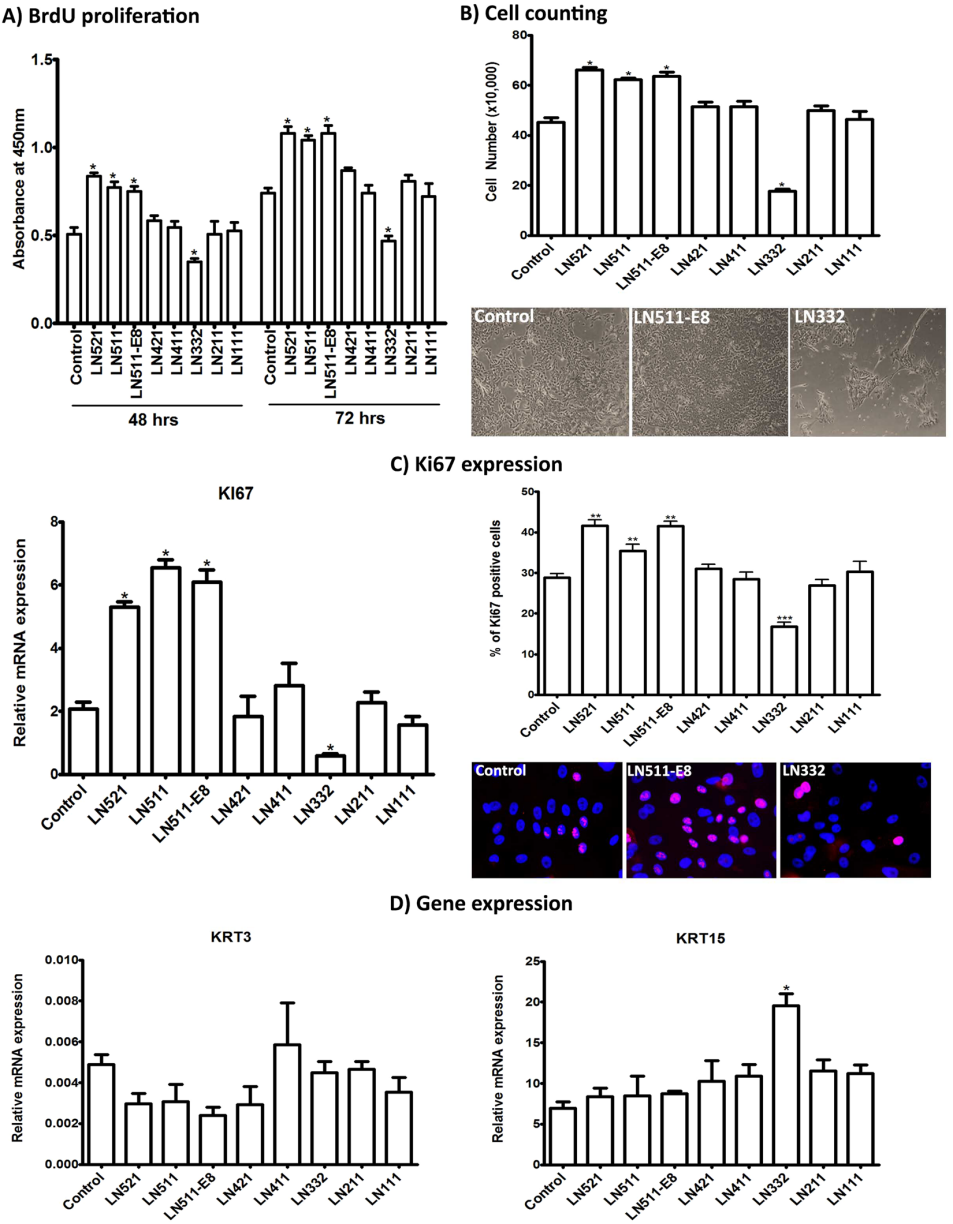
Effect of laminin isoforms on limbal progenitor cell proliferation and differentiation. (**A**) The effect of laminin (LN) isoforms on cell proliferation was tested by seeding limbal epithelial progenitor cells (LEPC) at a density of 15,000 cells/cm^2^ and spectrophotometric measurement of BrdU incorporation 48 and 72 hours after incubation. Data are expressed as means ± SEM (n = 5). (**B**) The effect of LN isoforms on LEPC proliferation was also analyzed by cell counting using CASY technology 7 days after seeding (15,000 cells/cm^2^); phase contrast images show LEPC cultured on tissue culture-treated plastic (control), LN-511-E8 and LN-332 before trypsinization for counting. Data are expressed as means ± SEM (n = 3). (**C**) The effect of LN isoforms on LEPC proliferation was additionally analyzed by Ki-67 expression on the mRNA level (left) and on the protein level (right). Ki-67 (KI67) mRNA levels were assessed by quantitative real-time polymerase chain reaction (qRT-PCR) primer assays, and data are expressed as means (2^−ΔCT^ × 1,000) ± SEM (*n* = 5). Ki-67 protein levels were monitored by counting the number of Ki-67-positive cell nuclei (magenta) and nuclei counterstained with DAPI (blue) using Cell F program (magnification ×200). Percentages (%) of positive cells are expressed as means ± SEM (n = 3). (**D**) The effect of LN isoforms on LEPC differentiation was analyzed by qRT-PCR primer assays of KRT3 and KRT15 expression levels. Data are expressed as means (2^−ΔCT^ × 1,000) ± SEM (*n* = 5); *p < 0.05; **p < 0.01; ***p < 0.001; Mann-Whitney *U* test.

The effect of LN isoforms on cell proliferation was assessed by BrdU incorporation assay 48 and 72 hours after seeding of LEPC on LN-coated culture wells (n = 5). Compared with uncoated controls, proliferation rates were significantly increased only in LEPC plated on LN-521 (1.7- and 1.4-fold), LN-511 (1.5- and 1.4-fold), and LN-511-E8 (1.5- and 1.4-fold) and were reduced in cells plated on LN-332 (−1.4- and −1.6-fold) (p = 0.02) (Fig. 4A). These findings were confirmed by cell counting (n = 3; p = 0.02) (Fig. 4B) and immunocytochemical staining for the proliferation marker Ki-67 (n = 3), which showed an increased percentage of positive cells on LN-521 (41.7 ± 2.5%; 1.4-fold; p = 0.001), LN-511 (36.4 ± 3.6%; 1.2-fold; p = 0.004), and LN-511-E8 (41.5 ± 2.5%; 1.4-fold; p = 0.009) but a decreased percentage of positive cells on LN-332 (16.7 ± 3.1%; −1.8-fold; p = 0.0007) compared to uncoated control (28.7 ± 2.8%) (Fig. 4C, right). Accordingly, mRNA expression levels of Ki-67 were also increased in LEPC cultured on LN-521 (2.6 ± 0.3-fold), LN-511 (3.1 ± 0.4-fold), and LN-511-E8 (2.8 ± 0.3-fold) and decreased in LEPC cultured in LN-332 (−4.1 ± 0.4-fold) compared to control (n = 5; p = 0.02) (Fig. 4C, left).

To assess a potential effect of LN isoforms on the differentiation state of LEPC, mRNA expression levels of KRT3, a cornea-specific differentiation marker, and KRT15, an established progenitor cell marker, were analyzed by qRT-PCR in LEPC passages 1 to 3 (n = 5). Although there was a trend towards suppression of KRT3 expression in LEPC cultivated on LN-521, -511, -511-E8, and -421 compared to control (Fig. 4D), differences were not statistically significant. Expression levels of KRT15 were also not significantly different from controls, except for LN-332, which supported maintenance of high KRT15 expression levels for up to 3 passages (p = 0.02).

These observations suggest that LN-332 supports an undifferentiated phenotype but inhibits proliferation, whereas LN-521, -511, and 511-E8 support cell proliferation without affecting differentiation, rendering LN-α5 containing isoforms as suitable candidates for tissue engineering purposes.

### Tissue engineering of corneal epithelial constructs

**Figure 5:**
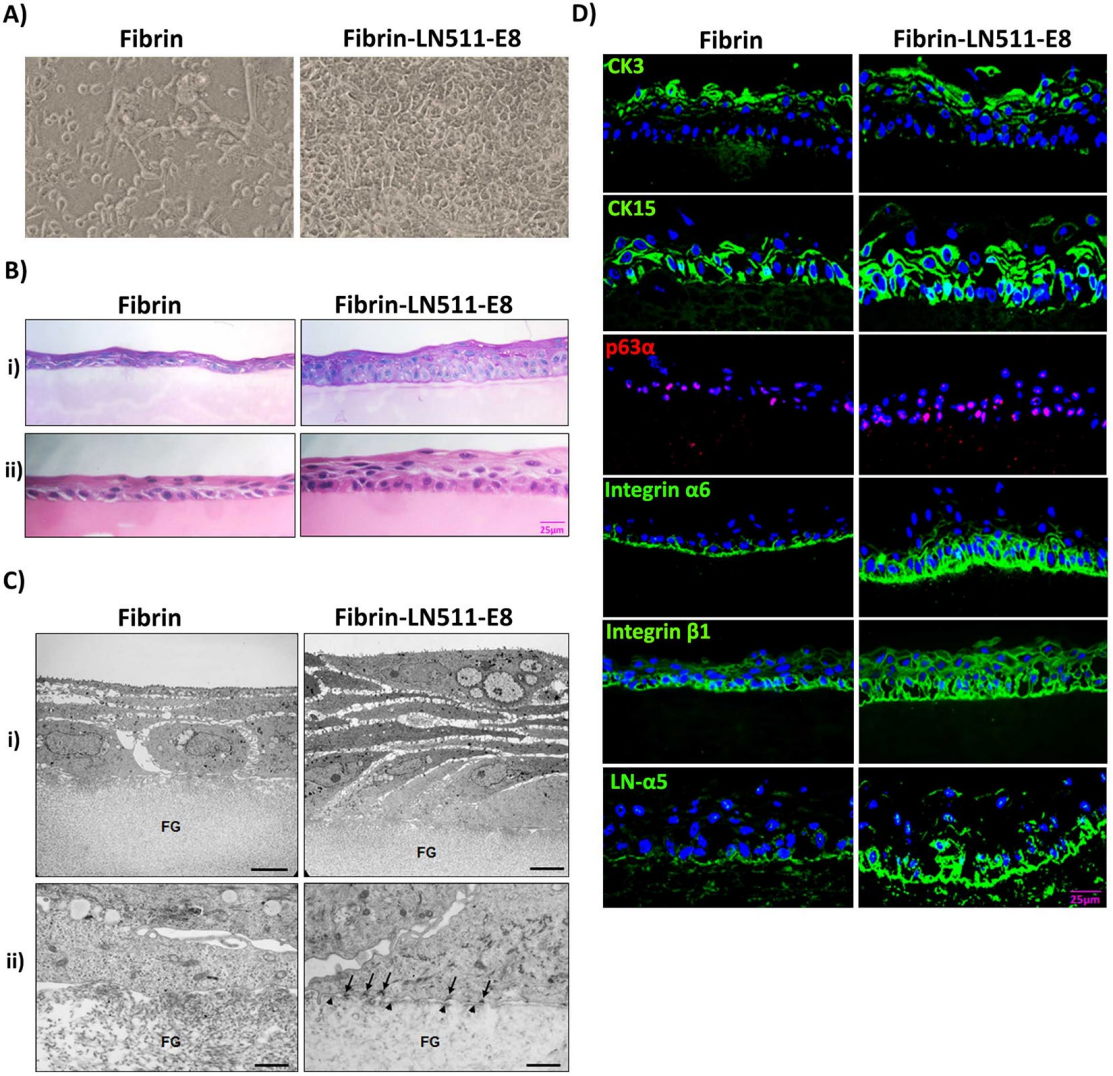
Tissue engineering of corneal epithelial constructs. (**A**) Phase contrast images of limbal epithelial progenitor cells (LEPC) growing on fibrin gels without or with incorporated recombinant laminin (LN)-511-E8 for 5 days (magnification ×100). (**B**) Light micrographs of epithelial cell sheets after two weeks of LEPC culture on fibrin gels without or with incorporated LN-511-E8 (i, periodic acid-Schiff staining; ii, hematoxylin-eosin staining; scale bar = 25 µm). (**C**) Transmission electron micrographs of epithelial cell sheets after two weeks of LEPC culture on fibrin gels (FG) without or with incorporated LN-511-E8; formation of hemidesmosomes (arrows) and basement membrane (arrowheads) can be seen on LN-511-E8 containing gels (scale bar = 5 µm in i, and 1 µm in ii). (**D**) Immunofluorescence analysis of epithelial constructs showing expression patterns of cytokeratin (CK)3, CK15, p63α, integrin α6, integrin ß1, and LN-α5 in epithelial constructs established on LN-511-E8 containing gels and bare fibrin gels (nuclear counterstaining with DAPI (blue); scale bar = 25 µm).

Since recombinant LN-E8 fragments have been shown to promote efficient and scalable culture of human stem cells under defined xeno-free conditions^41, 45^, we tested the applicability of LN-511-E8 for tissue engineering of corneal epithelial constructs (n = 5). To generate multilayered epithelial cell sheets on 3D-scaffolds suitable for clinical application, LEPC were seeded and cultivated for 12 to 14 days on fibrin gels prepared with or without incorporation of LN-511-E8 (10 µg/ml). LEPC expansion was markedly increased on LN-containing gels compared with untreated gels resulting in a confluent monolayer on LN-511-E8 by 5 days after seeding (Fig. 5A). Light microscopic analyses of tissue constructs showed a stratified epithelial cell sheet consisting of a cuboidal basal layer and 5 to 6 layers of flattened suprabasal cells on LN-511-E8 containing gels, but only 3 to 4 cell layers on LN-free gels (Fig. 5B). Transmission electron microscopy confirmed formation of well-organized multi-layered epithelial cell sheets consisting of a basal layer of cuboid cells covered by several layers of flattened elongated cells (Fig. 5C,i). Suprabasal cells showed typical ultrastructural signs of epithelial differentiation, such as desmosomes, cytoplasmic filaments, and apical microvilli. Basal cells appeared to be firmly attached to the LN-511-E8 containing fibrin gels by formation of hemidesmosomes and newly produced BM, whereas they appeared to only loosely adhere to LN-free fibrin gels where no BM deposition was evident (Fig. 5C,ii).

Immunofluorescence analysis of epithelial constructs showed expression of corneal epithelial differentiation marker CK3 in superficial layers and expression of progenitor cell markers CK15 and p63α in (supra)basal layers (Fig. 5D). The cell-matrix receptors α3, α6 and ß1 integrin could be mainly immunolocalized to the basal aspects of epithelial sheets, which also provided evidence of endogenous expression of LN-α5 and -α3. Protein expression was found to be generally more pronounced in epithelial constructs established on LN-511-E8 containing gels than on pure fibrin gels. These data suggest that LN-511-E8-coated fibrin gels promote *ex vivo*-expansion of LEPC and the generation of a stratified, firmly adherent, corneal epithelial-like cell sheet containing both differentiated and undifferentiated cells, which appears amenable for transplantation onto the corneal surface.

## Discussion

Using qPCR on microdissected LEPC clusters and confocal microscopy on tissue sections, we show that the BM of the limbal niche is enriched for LN chains α2, α3, α5, β1, β2, β3, γ1, and γ2, co-localizing with integrins α3, α6 and ß1 on the LEPC surface. Of note, mRNA expression of LN-γ3 could not be detected in LEPC, although LN-γ3 protein was clearly identified in the limbal zone by immunohistochemistry in this and previous studies^31, 46, 47^. In order to determine the role of LEPC in LN deposition within the limbal niche, we performed differential mRNA expression analyses in cultivated LEPC and supporting LMSC populations, both derived from LEPC clusters within the niche^32^. Our results clearly show the predominant expression of LN-α3 and –α5 by LEPC together with LN-β1, -β3, and -γ2 chains, suggesting the probable secretion of LN-332 and LN-511 by epithelial cells. By contrast, the LMSC preferentially expressed LN-α2 and -α4 chains together with -β2, -γ1 and -γ3 chains, and thus probably secrete and deposit LN-211/221 and LN-411/421/423 into the limbal BM. This differential expression pattern of laminin chains by epithelial and stromal fibroblast-like cells is consistent with findings in the epidermal BM, containing both epidermally derived LN-α3 and -α5 and mesenchymally derived LN-α2 and -α4, and suggests that, as in the skin, interaction between epithelial and mesenchymal cells is required to establish the limbal niche^48, 49^. The differential expression of LN-α5, but not LN-α3, by LEPC compared to BCEC further suggests that the LN-α5 chain may be an endogenous signature component of progenitor cells and may also present an appropriate exogenous cue for *ex vivo*-expansion of LEPC.

As a major component of BMs, LN isoforms are also enriched in other adult stem cell niches, where they regulate stem/progenitor cell function through interaction with cell surface receptors transducing molecular signals to the intracellular compartment^23–26, 50^. Consistent with the data presented here, interactions are predominantly mediated by one of the four LN-binding integrins, α3β1, α6β1, α6β4^43, 44^, and in the case of myogenic cells also α7β1, which activate specific signaling networks to modulate diverse cellular functions, such as adhesion, migration, proliferation, differentiation, stability of phenotype, and resistance towards apoptosis. The epidermal stem cell niche, for instance, contains LN-332 and LN-511, which interact with α6β4 and α3β1 integrins, respectively, and are hypothesized to maintain stem cell homeostasis through a precise ratio of LN-332 to LN-511^51–53^. Deposition of LN-332 and LN-511 gradients within the epidermal BM is regulated by integrin-linked kinase (ILK), which plays a crucial role in linking the ECM to the actin cytoskeleton of skin and hair follicle stem cells^52^. Detachment from LN-511/521-containing BMs has been considered a key factor in promoting epithelial differentiation. LN-332 and LN-511/521 have been also reported to be synthesized and secreted by human corneal epithelial cells *in vivo*, and to play a crucial role in the adhesion and migration of corneal epithelial *in vitro* ^54, 55^. Together with the data presented here, there is convincing evidence that both LN-332 and LN-511/521 through interaction with integrins α6β4 and α3β1/α6β1, respectively, form part of the limbal niche. However, whether the ratio between the two LN isoforms and their gradient of expression in the limbal BM determine LEPC function, requires further investigation. It also remains to be determined, whether ILK, which has been reported to transfer β1 integrin-mediated signals from the ECM to the Wnt/ß-catenin pathway in LEPC cultivated on amniotic membrane^56^, is a key regulator of the limbal niche ECM microenvironment.

Here, we further show that the different LN isoforms have different functions on LEPC *in vitro*. Whereas LN-511/521 supported integrin α3β1/α6β1-mediated LEPC adhesion, migration and proliferation without major effects on cellular differentiation, LN-332 promoted LEPC adhesion and migration, but suppressed proliferation, and maintained an undifferentiated phenotype, as reflected by high expression of the progenitor marker KRT15. All other LN isoforms tested, i.e. LN-421, -411, -211 and -111, showed no superior effects on adhesion and proliferation of LEPC compared to tissue culture control wells. These observations are different to the skin, where LN-α3 has been shown to promote and LN-α5 to inhibit keratinocyte proliferation^48^. However, in accordance with our observations, LN-511 and -521, which are also expressed in Descemet’s membrane and corneal endothelium, strongly supported *in vitro* adhesion and proliferation of human corneal endothelial cells^42^. Thus, the particular characteristics of LN-511/521, i.e. support of adhesion, proliferation and phenotype stability of corneal cells, render these isoforms best qualified for tissue engineering of LEPC-derived epithelial cell sheets without abandoning the progenitor phenotype. Indeed, recombinant LN-511 and -521 have been frequently used as substrates for efficient stem/progenitor cell expansion *in vitro* and have been shown to protect against apoptosis, to stimulate proliferation, and to maintain pluripotency and long-term self-renewal of both pluripotent and adult stem cells including human keratinocyte stem cells^57–60^.

Short recombinant E8 fragments derived from the long arm of LN have been reported as a viable and safe alternative to intact LN proteins in tissue engineering applications, because they do not involve the risk of contamination with xenogenic pathogens and immunogens^41^.These fragments have been shown to represent the minimum fraction conferring β1 integrin-binding activity and supporting efficient adhesion and proliferation of pluripotent stem cells in defined xeno-free media^45, 61, 62^. E8 fragments of LN-511 have also been used as a substrate for cultivation of human corneal endothelial cells with a similar efficacy to that obtained with full-length LN^42^. This could be confirmed by the present study, showing that LN-511-E8 fragments had similar potency in stimulating LEPC adhesion, migration, proliferation, and phenotype stability as intact LN-511. We further show that recombinant LN-511-E8 fragments incorporated into and on top of fibrin-based hydrogels improve *ex vivo*-expansion of LEPC and generation of a stratified epithelial cell sheet. The epithelial constructs generated on LN-511-E8 pre-functionalized scaffolds proved to be superior to those on bare scaffolds, displaying a well-organized stratified structure and firm attachment by α3β1 and α6β1 integrin-mediated anchorage to LN-α5 as well as preservation of a progenitor cell phenotype in (supra)basal layers and a differentiated phenotype in superficial layers.

In conclusion, *in vitro* presentation of appropriate ECM cues influencing stem cell function *in vivo* may represent a useful strategy to improve LEPC expansion and to maintain stem/progenitor cell function within the graft. The incorporation of chemically defined, bioactive, recombinant human E8 fragments of the limbal niche constituent LN-511 into clinically approved biosynthetic scaffolds like fibrin gels represents a promising avenue for safe, xeno-free corneal epithelial tissue engineering for ocular surface reconstruction. This defined culture technique may provide a significant improvement over current protocols for *ex vivo*-expansion of LEPC on undefined matrices, such as amniotic membrane or murine feeder cells, suffering from an intrinsic biologic variability, but this proposition has to be evaluated in preclinical *in vivo* studies. Future studies are also required to elucidate the nature of signaling pathways that are activated by LN-integrin interactions to determine LEPC function *in vivo* and *in vitro*.

## Methods

### Human tissues and study approval

Human donor corneas (n = 5 for LCM, n = 10 for immunohistochemistry) not suitable for transplantation with appropriate research consent were procured by the Erlangen Cornea Bank. In addition, organ-cultured corneoscleral tissue (n = 42) with appropriate research consent was provided by the Erlangen Cornea Bank after corneal endothelial transplantation. Informed consent to corneal tissue donation was obtained from the donors or their relatives. Experiments using human tissue samples were approved by the Institutional Review Board of the Medical Faculty of the University of Erlangen-Nürnberg (No. 4218-CH) and adhered to the tenets of the Declaration of Helsinki.

### Laser capture microdissection (LCM) and amplification of RNA

LCM and amplification of RNA was performed as previously described^32^. Briefly, corneal specimens destined for LCM were obtained from five donors (mean age, 69.6 ± 10.4 years) within 15 hours after death. After labeling of the superior, inferior, nasal, and temporal quadrants of donor globes, tissue sectors were embedded in optimal cutting temperature (OCT) compound (Tissue-Tek, Sakura Finetek Europe) and snap frozen in liquid nitrogen. Serial cryosections of 12 μm thickness were obtained under RNAse-free conditions from the superior or inferior quadrants, placed onto UV-irradiated (3000 mJ/cm^2^) PEN (polyethylene naphtalate) Membrane Slides (Carl Zeiss Microscopy, Göttingen, Germany), and stained with 0.01% cresyl violet. The PALM MicroBeam IV system (Carl Zeiss Microscopy) was used to isolate clusters of basal limbal epithelial cells (LEPC) and basal epithelial cells from central cornea (BCEC). From each donor eye, 100 cryosections were dissected (50 from the superior and 50 from the inferior quadrants, respectively) yielding a total of 500 cryosections used for sample collection.

RNA isolation from these specimens was achieved using the RNeasy Micro Kit (Qiagen, Hilden, Germany) including an on-column DNase digestion step according to the manufacturer’s instructions. Quality control was performed on a 2100 Agilent Bioanalyzer using the RNA 6000 Pico Kit (Agilent Technologies, Santa Clara, CA). Samples with an RNA concentration of 650–2,000 pg/µl and a RIN (RNA integrity number) of ≥7.0 were used for further analysis. Following RNA-amplification using the MessageAmp II aRNA Amplification Kit (Life Technologies GmbH, Darmstadt, Germany) according to the manufacturer’s protocol, aRNA (amplified RNA) concentration was measured on a Nanodrop ND1000 spectrophotometer (Thermo Scientific, Wilmington, DE) and quality control was again performed using Agilent technology.

### Real time RT-PCR

First-strand cDNA synthesis was performed using 5 µg of aRNA from tissue samples or RNA from cultured cells and Superscript II reverse transcriptase (Invitrogen, Karlsruhe, Germany) as previously described^32^. PCR reactions were run in triplicate in 1× TaqMan Probe Mastermix (Roche Diagnostics, Mannheim, Germany) or 1× SsoFast EvaGreen Supermix (Bio-Rad Laboratories, München, Germany), respectively, according to the manufacturers’ recommendations. Primer sequences (Eurofins, Anzing, Germany) are given in Table 2. For normalization of gene expression levels, ratios relative to the housekeeping gene GAPDH were calculated by the comparative *C*_T_ method (ΔΔ*C*_T_). Genes were considered as differentially expressed when their expression levels exceeded a two-fold difference in all specimens analyzed.

**Table 2:** Primers used in qRT-PCR primer assays.

Gene Symbol	Accession No.	Product Length	Probe No./SYBR	Sequence 5′–3′
CEACAM1	NM_001024912.2	113	P79	TCTTTGGGGCTAAGAGAAAGG
				GAGGTACCTGAGTATAGAGAACTCCAA
GAPDH	NM_002046.3	72	P3	CAGCAAGAGCACAAGAGGAA
				GTGGTGGGGGACTGAGTGT
ICAM1	NM_000201.2	73	P30	TTGACCTTTTGGGCTCAAGT
				TGGTGTTGTGAGCCTATGGT
Ki-67	NM_002417.4	78	P50	TTACAAGACTCGGTCCCTGAA
				TTGCTGTTCTGCCTCAGTCTT
KIT	NM_000222.2	61	P6	GAGTAGCTTACCAGAAGCTTCCATAG
				CATAGGGACTGATGCCTTCC
KRT3	NM_057088.2	92	P74	CCTGTGATTGTCCAGGTGTG
				ATACATCAGAGCTGTAGTGAGCATC
KRT15	NM_002275.3	60	P10	CATTGGCATCAGGGAAGC
				TTGATGTGGAAATTGCTGCT
LAMA1	NM_005559.3	149	SYBR	CTGGGACCGAGTCCTGAAC
				ACACAACATCTCTCCCCAGAA
LAMA1	NM_005559.3	60	P38	AGGATGACCTCCATTCTGACTT
				CCTTACATGGGCACTGACCT
LAMA2	NM_000426.3	68	P81	GCAAGCCACTGGAGGTTAAT
				TGGGCATGATACAGGTTGAA
LAMA3	NM_198129.1	72	SYBR	GCACTGATTACCCAATGCACC
				ACCTGTCTGGTTTGTGTCCTG
LAMA3	NM_198129.1	65	P29	CCAGGAATATGGGTTGCTTG
				GGGAGCAGCACCAGGTAAT
LAMA4	NM_001105209.1	68	P55	GCCACACTCGTCCTTCTCTC
				CCCAGGTGAAACTCTCAAGG
LAMA5	NM_005560.3	75	P44	ATGACTCGCTCTGTGGAGGT
				GGGGTTGGCTGTGTCCTA
LAMB1	NM_002291.2	72	P82	AAGCCAGAAAGTTGCTGTGTATAG
				CCTTGTTCACCTCAGCCATT
LAMB2	NM_002292.3	100	P43	AAGGCCTACCCCAGTTCCTA
				GGGTTCACACTGGTTTATTGG
LAMB3	NM_001127641.1	72	P23	GGCATGCCATTGAAACTAAGA
				AGAACTAAAGGCGGGGGATA
LAMB4	NM_007356.2	67	P5	CCCCACACCCTGTCCTTATT
				ATTTTCCTGGTGGCATTTCA
LAMC1	NM_002293.3	92	P66	GCCATTATTTTATTGTCTAGCTCCA
				ATCCCTGTGTCAACCAGCAT
LAMC2	NM_005562.2	96	P65	CACTCTGTGCCTTTCTACAACTG
				CCAAGGTGGAAGTGCCTCT
LAMC3	NM_006059.3	67	P14	CAGGACTCCTCAGCATTTCC
				TTGCCATCTGCTGGAAGAG
SOX2	NM_003106.3	85	P65	GGGGGAATGGACCTTGTATAG
				GCAAAGCTCCTACCGTACCA

Primers were used for Probe based (Universal Probe Library) or SYBR Green based qRT-PCR assays with an annealing temperature of 60 °C.

### Immunohisto- and immunocytochemistry

Corneoscleral tissue samples obtained from 10 normal human donor eyes (mean age, 72.3 ± 11.6 years) and 3D-cultures were embedded in optimal cutting temperature (OCT) compound and frozen in isopentane-cooled liquid nitrogen. Cryosections of 4 μm thickness were cut from the superior or inferior quadrants, fixed in cold acetone for 10 minutes, blocked with 10% normal goat serum, and incubated in primary antibodies (Table 1) diluted in PBS overnight at 4 °C. Antibody binding was detected by Alexa 488-conjugated secondary antibodies (Molecular Probes, Eugene, OR) and nuclear counterstaining was performed with DAPI (Sigma-Aldrich, St. Louis, Missouri). Immunolabelled cryosections and cultured LEPC were examined with a fluorescence microscope (Olympus BX51; Olympus, Hamburg, Germany) or a laser scanning confocal microscope (LSM 780; Carl Zeiss Microscopy). In negative control experiments, the primary antibodies were replaced by PBS or equimolar concentrations of an irrelevant isotypic primary antibody.

### Cell culture

LEPC clusters were isolated from 42 corneoscleral buttons (mean age, 69.2 ± 7.1 years) as previously described^32^. Briefly, organ-cultured corneoscleral tissue with appropriate research consent was provided by the Erlangen Cornea Bank after corneal endothelial transplantation. After rinsing in Hanks’ balanced salt solution, the tissues were cut into 12 one-clock-hour sectors, from which limbal segments were obtained by incisions made at 1 mm before and beyond the anatomical limbus. Each limbal segment was enzymatically digested with 2 mg/mL collagenase A at 37 °C for 18 hours to generate epithelial-mesenchymal stem/progenitor cell clusters. Cell clusters were isolated from single cells by using reversible cell strainers with a pore size of 37 µm (Stem Cell Technologies, Köln, Germany). Isolated cell clusters were further dissociated into single cells by digestion with 0.25% trypsin and 0.02% EDTA (Pan Biotech, Aidenbach, Germany) at 37 °C for 10–15 min. Single cell suspensions were seeded into T75 flasks (Corning, Tewksbury, MA) in Keratinocyte serum free medium (KSFM) supplemented with bovine pituitary extract, epidermal growth factor (Life Technologies) and 1× penicillin-streptomycin-amphotericin B mix (Pan Biotech) to enrich epithelial cell population (LEPC) and the flasks were incubated at 37 °C under 5% CO_2_ and 95% humidity. To enrich the associated mesenchymal stromal cells (LMSC), single cell suspensions were seeded into T75 flasks in Mesencult media (Stem Cell Technologies) and incubated at 37 °C under 5% CO_2_ and 95% humidity. The media was changed every second day. Cultivated cells were used for real time PCR (n = 10), flow cytometry (n = 3), functional assays (adhesion: n = 4, integrin blocking: n = 4, migration: n = 3, proliferation: n = 13) and tissue engineering (n = 5).

### Flow cytometry

LEPC cluster derived and enriched mesenchymal cells (LMSC, P1) were isolated from 3 organ-cultured corneoscleral buttons (mean age, 66.5 ± 4.6 years) and characterized by flow cytometry using anti-CD90, -CD73, -CD105, -CD44, -CD34, -CD45, -CD31 and isotype control antibodies (BD Biosciences, Heidelberg, Germany). LEPC were characterized using anti-α3 integrin-APC, anti-α6 integrin-APC, anti-β1 integrin-FITC and anti-β4 integrin-eFluor660 antibodies (eBioscience, Frankfurt, Germany). Single cell suspensions (0.5–1 × 10^6^ cells) were incubated with saturating concentrations of respective primary antibodies or conjugated antibodies in 100 µl PBS, 0.1% sodium azide and 2% fetal calf serum for 40 min. After three washes, the cells were centrifuged at 200 × g for 5 min. Primary antibody reactions were incubated with Alexa Fluor 488-conjugated goat anti-mouse IgG for 30 min. Cells were then washed and re-suspended in ice-cold PBS. After addition of 5 µl of 7-amino-actinomycin D (7-AAD) to exclude dead cells, cytometry was performed on a FACSCanto II (BD Biosciences) by using FACS Diva Software. A total of 10,000 events were acquired to determine the positivity of cell surface markers.

### Cell adhesion assay

LEPC isolated from 4 organ-cultured corneoscleral buttons (mean age, 74.2 ± 5.8 years) were seeded onto 96 well-plates coated with recombinant LN-111, -211, -332, -411, -421, -511, -521 (1.0 µg/cm^2^; BioLamina, Sundbyberg, Sweden) and recombinant LN-511-E8 (0.5 µg/cm^2^; Nippi, Tokyo, Japan) as per manufacturers’ recommendations. Coating solutions were prepared in 1x DPBS (Dulbecco’s phosphate-buffered saline) with calcium and magnesium and incubated in culture wells either for two hours at 37 °C (fast coating) or overnight at 4 °C (slow coating) as per manufacturer’s instructions. Cells (P1-P2) were seeded at a density of 50,000 cells/cm^2^ and left to adhere for 30 and 60 min at 37 °C. Standard tissue culture treated plates were used as control. After incubation, plates were washed with DPBS using a Static Cell Adhesion Wash Chamber (Glycotec, Maryland, USA) to remove non-adherent cells, and the adherent cells were fixed with 4% paraformaldehyde/PBS for 15 min and stained with 0.1% crystal violet for 20 min. After three washes with water, stained cells were extracted with 1% sodium dodecyl sulfate and quantified by measuring optical density (OD) at 570 nm using a spectrophotometer (Multiskan Spectrum; Thermo Scientific, Waltham, USA). All experiments (n = 4) were performed in quadruplicates. The fold change values were calculated as OD of the LN/OD of control.

The effect of an interaction between cellular integrins and extracellular LNs on cell adhesion was evaluated by seeding LEPC (50,000 cells/cm^2^) in 96-well plates coated with recombinant LN-332, -511, -521, and LN-511-E8 in the presence or absence of integrin-neutralizing antibodies, i.e., anti-α3 integrin (20 µg/ml) (Merck Millipore, Darmstadt, Germany), anti-α6 integrin (20 µg/ml) (Merck Millipore) and anti-β1 integrin (2.5 µg/ml) (R&D Systems Inc., Minneapolis, MN, USA). 60 minutes after seeding, the numbers of adherent cells were determined as described above. LEPC used for integrin blocking assays were isolated from 4 organ-cultured corneoscleral buttons (mean age, 67.7 ± 6.1 years) and all experiments were performed in quadruplicates.

### Cell migration assay

To exactly measure the change in the cell-covered area over time, 2 well-culture inserts with a defined cell-free gap were used (ibidi GmbH, Planegg, Germany). Briefly, the wells were coated with LN isoforms as described above and seeded with 70 µl of a LEPC suspension containing 5 × 10^5^ cells/ml. LEPC were isolated from 3 organ-cultured corneoscleral buttons (mean age, 71.3 ± 6.1 years). After formation of a cellular monolayer (24 hours), the silicone inserts were removed and the culture medium was supplemented with 2.5 µg/ml of soluble LNs. Images of each well were acquired immediately following insert removal (0 hours) and after 3 and 6 hours by using an inverted microscope (CKX41, Olympus). Image analysis software Cell F (Olympus) was used to measure areas that were free of migrating cells. All experiments (n = 3) were performed in triplicates.

### Cell proliferation assays

LEPC used for cell proliferation assays were isolated from a total of 13 corneoscleral buttons after corneal endothelial transplantation (mean age, 70.0 ± 8.0 years).The effect of LNs on LEPC proliferation was quantified using the Cell Proliferation ELISA BrdU Colorimetric Assay Kit (Roche Diagnostics). Cells (P1-P2) were seeded into 96-well plates, coated with LN isoforms as described above, at a density of 5000 cells/well, cultured for 48 and 72 hours, and labeled with BrdU according to the manufacturer’s instructions. Absorbance was measured at 450 nm using a spectrophotometer (Multiskan Spectrum), and fold change values were calculated as described above. Experiments (n = 5) were performed in quadruplicates.

For immunocytochemical analysis of cell proliferation, LEPC (n = 3) were seeded at a density of 10,000 cells/well into 4 well- glass chamber slides (LabTek; Nunc, Wiesbaden, Germany), cultured for 72 hours, and stained with antibodies against Ki-67 (Abcam; Cambridge, UK). Ki-67 positive cells were counted in the 4 wells using Cell^F^ image analysis software (Olympus). Ki-67 expression was also analyzed by real time PCR as described above (n = 5).

In addition, cell numbers were counted after trypsinization of LEPC 7 days after seeding (5 × 10^4^ cells/well) in 6-well culture plates using CASY technology (Schärfe System GmbH, Reutlingen, Germany) (n = 3).

### Tissue engineering of epithelial constructs

Scaffolds for tissue engineering and 3D-cell culture were prepared from fibrin as previously described^38^. Briefly, the gels were prepared by dissolving fibrinogen and thrombin stock solutions (Tisseel; Baxter Deutschland GmbH, Unterschleißheim, Germany) in 1.1% NaCl and 1 mM CaCl_2_ to a final concentration of 10 mg/ml fibrinogen and 3 IU/ml thrombin. Recombinant LN-511-E8 (10 µg/ml) was incorporated into the gels, which were placed into 24 well-culture inserts and allowed to polymerize at 37 °C. After washing with PBS, gels were additionally coated with LN-511-E8 (5 µg/ml) overnight. LN-511-E8 free gels served as controls. LEPC (P1) were isolated from 5 organ-cultured corneoscleral buttons (mean age, 65.0 ± 7.5 years) and seeded onto coated and uncoated control gels at a density of 1 × 10^5^ cells/cm^2^ and cultivated in KSFM media under low calcium concentrations (0.09 mM Ca^2+^) for 5 to 6 days. Culture conditions were switched to DMEM/Ham’s F12 (Hyclone; GE Health Care Life Sciences, Freiburg, Germany) supplemented with Human Corneal Growth Supplement (Life Technologies), 10% FCS (GE Health Care Life Sciences), and high calcium concentrations (1.2 mM Ca^2+^) in order to promote cell differentiation. After another 6 to 8 days of cultivation, gels were fixed for immunohistochemistry as described above and for light and electron microscopy.

For light and electron microscopy, gels were fixed in 2.5% glutaraldehyde in 0.1 M phosphate buffer, dehydrated, and embedded in paraffin or epoxy resin, respectively, according to standard protocols. Paraffin sections were stained with hematoxylin and eosin, and ultrathin sections were stained with uranyl acetate-lead citrate and examined with an electron microscope (EM 906E; Carl Zeiss Microscopy).

### Statistical analysis

Statistical analyses were performed using the GraphPad InStat statistical package for Windows (Version 5.04; Graphpad Software Inc., La Jolla, CA). Data are expressed as mean ± standard error of the mean from individual experiments. The Mann-Whitney *U* test was performed to assess statistical significance. A *p* value of <0.05 was considered statistically significant.

### Data availability

Any additional data beyond those included in the main text that support the findings of this study are also available from the corresponding author upon request.
